# Administrative, legal, and organizational hurdles in data linkage: Experiences and lessons from the recent National Center for Health Statistics (NCHS) - U.S. Department of Housing and Urban Development (HUD) data linkage project

**DOI:** 10.23889/ijpds.v1i1.351

**Published:** 2017-04-19

**Authors:** Jon Sperling, Patricia Lloyd, Veronica Helms, James Brittain

**Affiliations:** 1 US Department of Housing and Urban Development / Office of Policy Development and Research; 2 Centers for Disease Control and Prevention / National Center for Health Statistics

## Objectives

Through a collaborative agreement between the NCHS and HUD, the first linkage of two national population health surveys to HUD administrative records of housing assistance was recently completed. Based on this multi-year effort, this paper emphasizes key requirements for making progress in and between regulatory and legal environments; confidentiality and data security.

## Background

As the principal health statistics agency in the United States, the National Center for Health Statistics (NCHS) collects and disseminates statistical information that guide actions and policies to improve the health of the American people. The mission of the U.S. Department of Housing and Urban Development (HUD) is to create strong, sustainable, inclusive communities and quality affordable homes for all. Prior to the release of the NCHS-HUD linked file, there was no existing data source to reliably estimate the prevalence of health conditions and outcomes for households receiving HUD housing assistance. Rather than conducting its own health survey, costing millions of dollars, HUD leveraged the existing NCHS and Census knowledge base, the data linking expertise of NCHS and its Research Data Center (RDC) infrastructure. With the NCHS-HUD linked data files, analysts and researchers can now examine health conditions, health behaviors, access to health services and use of health services in relation to housing.

## Approach

While technical (e.g., temporal, spatial, definitional, linkage keys) challenges for linking national-level surveys and administrative data have been the subjects of many discussions, often the organizational, legal and behavioral issues are more daunting and usually determine success. To comply with regulations at NCHS and HUD, a memorandum of understanding clearly outlined the manner with which personally identifying information (PII) would be protected, and how data would be shared across agencies. 

## Results and Conclusion

Through this extraordinary and innovative, no-cost, collaborative and strategic research partnership with the Department of Health and Human Services/Centers for Disease Control and Prevention/NCHS, HUD is learning more about the health of tenants receiving housing assistance. The data linkage project is a model for data sharing that aligns with U.S. Office of Management and Budget (OMB) directives for sharing data across federal agencies to reduce respondent burden and improve federal programs and as well as the health of American people. 

